# Corrosion protection of polypyrrole films doped with 3-nitrosalicylate on zinc substrates

**DOI:** 10.1039/d6ra05088c

**Published:** 2026-08-03

**Authors:** Tran Minh Thi, Ha Manh Hung, Le Van Khoe, Le Minh Duc, Nguyen Dang Dat, Vu Thi Huong, Nguyen Thi Bich Viet, Ngo Xuan Luong, Doan Thi Yen Oanh, Vu Quoc Trung

**Affiliations:** a Institute of Theoretical and Applied Research, Duy Tan University Hanoi 100000 Vietnam; b Faculty of Nature Science, Duy Tan University Da Nang 550000 Vietnam; c Faculty of General Education, Hanoi University of Mining and Geology Dong Ngac Hanoi 100000 Vietnam; d Faculty of Natural Sciences, Hong Duc University 565 Quang Trung, Hac Thanh Thanh Hoa 40100 Vietnam; e Branch of National Institute of Occupational Safety and Health & Environmental Protection in Central of Vietnam 178 Trieu Nu Vuong, Hai Chau Da Nang 540000 Vietnam; f Hanoi National University of Education 136 Xuan Thuy, Cau Giay Hanoi 100000 Vietnam trungvq@hnue.edu.vn; g Publishing House for Science and Technology, Vietnam Academy of Science and Technology Nghia Do Hanoi 100000 Vietnam

## Abstract

Using 3-nitrosalicylic acid (3Nisa) as the dopant and pyrrole as the monomer, Ppy/3Nisa coatings were successfully electrodeposited on zinc substrates by a simple one-step electrochemical process. SEM analysis and FT-IR and Raman spectroscopy confirmed the successful formation of compact and homogeneous Ppy/3Nisa films on the Zn surface. Electrochemical studies demonstrated that the incorporation of 3Nisa significantly improved the corrosion resistance of Zn in a 3.5 wt% NaCl solution. The optimized coating (Z3) exhibited the lowest corrosion current density of 1.47 µA cm^−2^ and a corrosion inhibition efficiency of 98.6%. Open circuit potential measurements showed that the coated Zn substrates remained in the passive region for extended immersion times, while cyclic voltammetry revealed the stable redox behavior of the Ppy/3Nisa films, lasting for approximately 500 h. Electrochemical impedance spectroscopy and salt spray tests further confirmed the enhanced barrier properties and structural stability of these coatings. The improved anticorrosion performance was attributed to the dense coating structure, Zn–N/Zn–O interactions, and controlled release of 3Nisa dopant anions, which promoted the self-healing and *in situ* repassivation of the Zn surface. These findings suggest that Ppy/3Nisa coatings are promising materials for corrosion protection and potential Zn-based energy storage applications.

## Abstract 2

A compact Ppy/3-nitrosalicylate coating was electrodeposited on Zn, achieving 98.6% corrosion inhibition through enhanced barrier protection, Zn–N/Zn–O interactions, and inhibitor-assisted repassivation.

## Graphical abstract

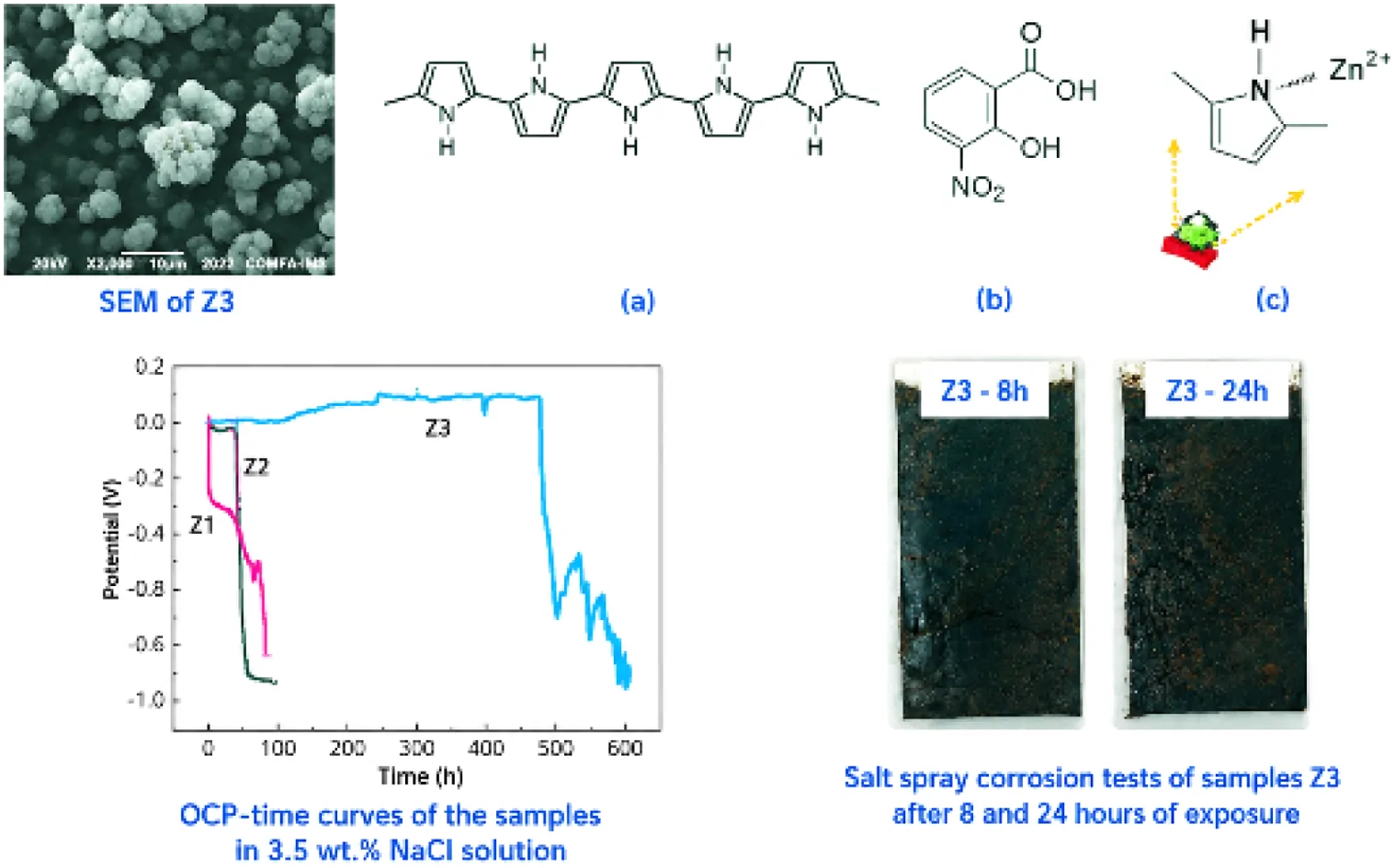

## 1 Introduction

The corrosion and protection mechanisms of metals and alloys are big challenges in various fields that need to be addressed. Some of the intrinsically conducting polymers (ICPs) have attracted significant attention in basic and applied research due to their corrosion resistance and electrochemical properties.^1–6^ These materials show strong potential for applications in batteries, supercapacitors, electrocatalysis, biosensors, electromagnetic shielding, and anticorrosion coatings. Among the most promising ICPs are polyaniline, polypyrrole (Ppy),^5–12^ poly(ethylenedioxythiophene) (PEDOT), polythiophene and their derivatives, in which Ppy has gained particular interest due to its excellent energy storage capacity, simple synthesis, good environmental stability, high electrical conductivity, and relatively low cost compared with other ICPs.^13–18^ Several previous publications have focused on the role and corrosion resistance of composite materials containing Ppy and some metals, such as Zn, Cu, Al–Zn, molybdate, Ti, and 304 stainless steels.^19–29^

Recently, many publications have shown the utmost importance of the dopant anions of Ppy. Some properties of Ppy can be improved by changing different dopant anions. Many types of metal nanocomposites (Ni, Fe, Al, Cu, Zn, Ti,… and their alloys) and polymers (polyaniline, polystyrene, chitosan, epoxy, polyurethane, and polypyrrole) with different fabrication methods have been studied (electrochemical method, electrochemical deposition, electropolymerization, drop casting technique, galvanostatic electrodepositions, pulse current deposition, and template *in situ* polymerization).^1,17,18,30,31^

Numerous corrosion-protection mechanisms have been proposed for conducting polymer coatings, including anodic passivation, barrier protection, deoxygenation effects, controlled inhibitor release, self-healing behavior, and interfacial coordination interactions. The dominant mechanism depends on the type of conducting polymer, dopant, substrate, and exposure environment. Application studies on energy storage batteries containing polyaniline, polypyrrole, Zn, Cu, and microcrystalline cellulose (MCC) have also been conducted.^2,4–7,9–12,32^ Recent research studies have focused on studying polypyrrole (Ppy) prepared on low-carbon steel by electrochemical polymerization in a solution containing a pyrrole monomer and succinic acid.^33–36^ The mechanism and corrosion behavior of low-carbon steel (CT3) with a Ppy film in a 3% NaCl solution,^33^ GO/Mo/Sal (graphene, molybdate and salicylate),^34^ CT3 mild steel^35^ and low carbon steel^36,37^ have been studied. Due to the environmental friendliness and superior advantages of Ppy, studies that use Ppy and additives to protect metals and alloys in an application-oriented manner are of interest. The trend of expanding anti-corrosion, anti-oxidation, bio-friendly, and environmentally friendly applications^38,39^ in energy storage, such as using Zn-doped Ppy batteries,^40–43^ is attracting attention alongside research on the bonding between ZnO and Ppy, the corrosion behavior of Zn coatings^44,45^ and the corrosion of polypyrrole-coated metals.^46^ However, the quality and effectiveness of corrosion protection depend on the type of conductive polymer, additives, metal or alloy requiring corrosion protection, and conditions and manufacturing methods. Research results using Ppy, additives, and manufacturing methods to protect metals and alloys are abundant and achieve very different criteria but have not received much attention.

In recent years, the corrosion-protection performance of polypyrrole coatings has been shown to depend strongly on the nature of the incorporated dopant anions since these species not only maintain charge balance during electropolymerization but may also affect film compactness, interfacial adhesion, ion transport, and inhibitor-release behavior.^6–8^ Various organic dopants such as salicylates, sulfonates, and carboxylates have been explored in Ppy-based anticorrosion systems.^34,35^ However, the effect of nitro-substituted salicylate dopants on Zn protection has not been systematically clarified. In particular, 3-nitrosalicylic acid (3Nisa) is an interesting candidate because it combines carboxylate functionality, oxygen-containing donor groups, and a nitro substituent that may alter the electron distribution of the aromatic framework and enhance its interaction with Zn or Zn^2+^ species. Such a molecular structure suggests that 3Nisa may play a dual role in Ppy coating: as a dopant anion for polymer formation and as an inhibitory species capable of contributing to interfacial stabilization through Zn–O/Zn–N interactions and inhibitor-assisted repassivation. Nevertheless, the electropolymerization of Ppy/3Nisa on active Zn substrates and the corresponding concentration-dependent anticorrosion behavior in chloride-containing media have not been systematically investigated. Therefore, the present work aims to clarify whether 3Nisa-doped Ppy coatings can improve the corrosion resistance of Zn through a combination of barrier effects, coordination-assisted stabilization, and electrochemically active inhibitor functionality rather than cathodic protection.

In this work, homogeneous Ppy with 3-nitrosalicylic acid (3Nisa) doping coatings was successfully electrodeposited on zinc substrates. The one-step electropolymerization process was carried out in an aqueous medium. The corrosion protection efficacy of the Ppy/3Nisa films was systematically studied through surface analysis of SEM images, FT-IR spectral analysis, Raman spectroscopy, potential–time curves, CV curves, Bode plots, Tafel plots, and the time stability of the samples. The electrochemical corrosion protection mechanism was also discussed.

## 2 Materials and methods

### 2.1. Materials

All chemicals used in this study, including pyrrole monomer (98%), 3-nitrosalicylic acid (99.5%), and sodium chloride (99.5%), were purchased from Sigma-Aldrich. Pyrrole monomer was distilled carefully and stored in a dark atmosphere at a low temperature before use.

Pure Zn samples (Vietnam) of dimensions 4 cm × 2 cm × 0.5 cm were used as substrates for polymer coatings.

### 2.2. Preparation of Zn samples

Prior to each experiment, the Zn substrates were mechanically polished with 2000-grit emery paper to obtain a reproducible surface finish. After polishing, the samples were thoroughly rinsed with distilled water to remove abrasive residues, washed with ethanol to eliminate organic contaminants, and finally dried under a nitrogen stream. The cleaned Zn substrates were used immediately for electropolymerization in order to minimize surface oxidation before coating deposition.

### 2.3. Electrosynthesis of the Ppy/3Nisa film on Zn

Ppy/3Nisa films were prepared by galvanostatic electropolymerization of pyrrole in aqueous 3Nisa-containing electrolytes. Pyrrole monomer (0.10 M) was first dispersed in distilled water by sonication for 30 min to obtain a homogeneous solution. Subsequently, 3Nisa was added at different concentrations listed in Table 1 (0.005, 0.01, and 0.02 M for Z1, Z2, and Z3, respectively), and the resulting solutions were magnetically stirred for 60 min before deposition. Electropolymerization was carried out at a constant current density of 2.2 mA cm^−2^ using a conventional three-electrode cell, with Zn as the working electrode, Ag/AgCl as the reference electrode, and a Pt sheet as the counter electrode. The electrolytes were continuously stirred during deposition to maintain concentration homogeneity.

**Table 1:** Samples of the Ppy/3Nisa coating on Zn

No.	Sample	Pyrrole (mol L^−1^)	3Nisa (mol L^−1^)
1	Z1	0.10	0.005
2	Z2	0.10	0.01
3	Z3	0.10	0.02

### 2.4. Characterization of the coating

The synthesized nanocomposite coatings were characterized by several physicochemical methods. The surface morphology of the coatings was examined by field-emission scanning electron microscopy (FESEM, Hitachi S-4800). Fourier transform infrared (FT-IR) spectra were recorded using a Shimadzu Prestige-21 spectrometer in the wavenumber range of 500–4000 cm^−1^. Raman spectra were obtained using a Raman spectrometer (D8 Advance, Bruker, Germany).

### 2.5. Corrosion test

The anticorrosion performance of the Ppy/3Nisa-coated Zn substrates was evaluated by open circuit potential (OCP), electrochemical impedance spectroscopy (EIS), and Tafel polarization measurements in an aerated 3.5 wt% NaCl solution at room temperature using a Zahner electrochemical workstation (Germany). EIS measurements were carried out over a frequency range of 10 mHz to 100 kHz with a small sinusoidal perturbation amplitude. Salt spray tests were performed to investigate the corrosion resistance of the coatings under aggressive saline conditions.

Cyclic voltammetry (CV) measurements were conducted in a 0.1 M tetrabutylammonium bromide (TBA-Br) solution to favor anion doping while suppressing cation incorporation into the polymer matrix.

All electrochemical experiments were performed using a conventional three-electrode cell configuration. An Ag/AgCl electrode and platinum sheet were employed as the reference and counter electrodes, respectively.

## 3 Results and discussions

### 3.1. Electropolymerization of Ppy/3Nisa on Zn substrates

**Fig. 1:**
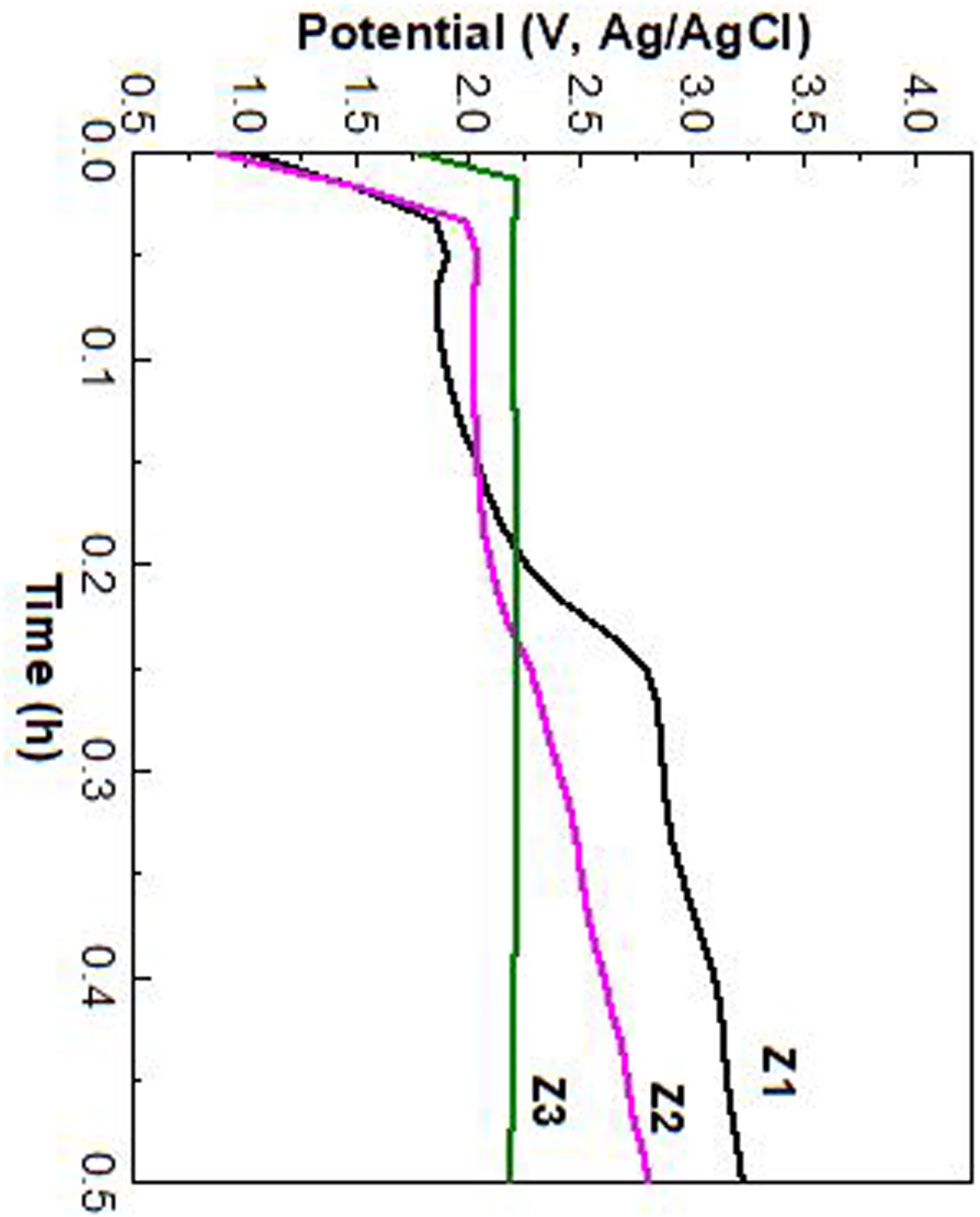
Potential-time curves of Ppy/3Nisa formation on Zn.

The electrosynthesis of Ppy/3Nisa films on zinc substrates can also be performed by the galvanostatic technique in a one-step procedure with solutions of different 3Nisa concentrations. The potential–time curves are presented in Fig. 1.

The curves can be divided into two stages. In the first stage (about 3 minutes), the potential increased due to passive layer formation and polymer nucleation. In the second stage, the potential stabilized at a constant value over time (sample Z3). Samples Z1 and Z2 showed increasing potential continuously. It should be noted that the growth of oxide and Ppy/3Nisa on the Zn occurred strongly.

The potential stability of sample Z3 over time is noteworthy, demonstrating that the 3Nisa-induced passive film on Zn was sufficiently stable and robust to sustain pyrrole oxidation. With an optimal 3Nisa concentration of 0.02 M, the film-coated Zn behaved similarly to that of the inert electrode. The positive voltage (2.2 V) on the Z3 model remains stable for a long time, while studies only achieve (0.1–0.19 V) on low-carbon steel,^34,35^ demonstrating the sustainability of the manufactured model.

### 3.2. Characterization

#### 3.2.1. Morphological characterization by SEM

**Fig. 2:**
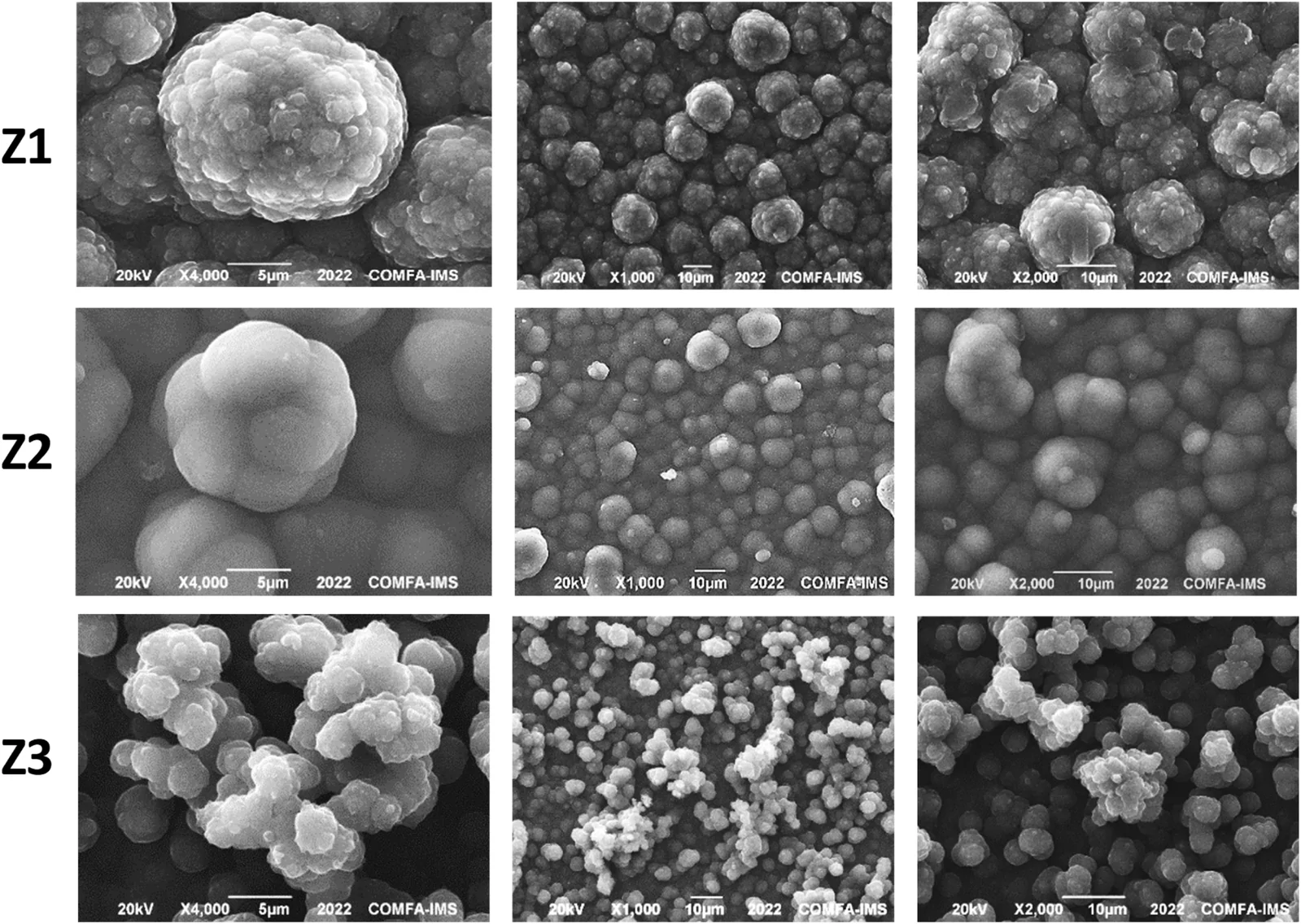
SEM images of the Z1, Z2, and Z3 samples under different magnifications.

Fig. 2 presents the SEM images of samples Z1, Z2, and Z3 at different magnifications. The SEM micrographs reveal significant differences in the surface morphologies of the three samples. For sample Z1, the Ppy/3Nisa particles exhibit a relatively spherical and well-defined morphology. The particle size distribution is comparatively narrow, with most particles having diameters in the range of 10–20 µm. Although the particles are densely packed, their individual boundaries remain distinguishable. In addition, the particle surfaces appear rough and nodular, indicating secondary growth or the aggregation of smaller crystallites.

In contrast, sample Z2 exhibits particles with more uniform shapes and sizes, leading to a narrower size distribution. Significant agglomeration is observed, resulting in irregular clusters rather than discrete spherical particles. The average particle size is somewhat smaller than that of Z1, ranging from 10 to 15 µm; notably, the particles are fused together, suggesting enhanced interparticle interactions. Sample Z3 clearly displays a characteristic cauliflower-like or globular morphology, which is typical of electrochemically synthesized Ppy/3Nisa.

These aggregates, with sizes ranging from 5–10 µm, are formed through the coalescence of nanosized primary particles, giving rise to a hierarchical and highly rough surface structure. The film appears highly porous, containing interconnected clusters and voids between the agglomerates. Although the individual globules are generally submicron- to micron-sized, they are composed of much smaller primary building blocks. This morphology indicates the formation of a continuous but non-smooth film, which may provide a high surface area and abundant active sites.

#### 3.2.2. Structural characterization by FT-IR and Raman spectroscopy

**Fig. 3:**
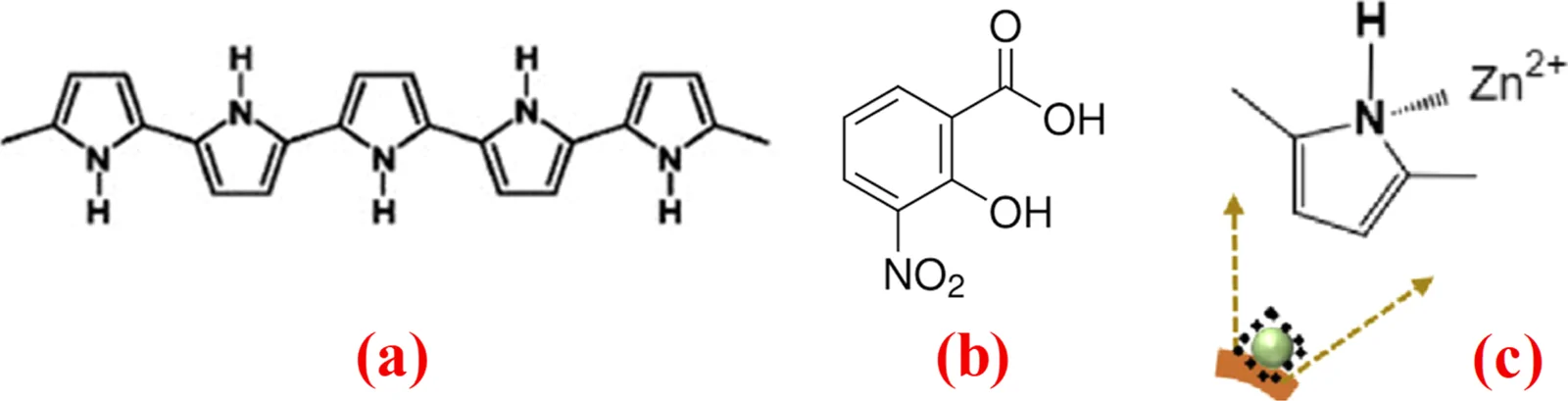
Chemical structures of (a) Ppy and (b) 3Nisa. (c) Schematic of the Ppy coating mechanism on Zn.

3Nisa (C_7_H_5_NO_5_) contains a strongly electron-withdrawing –NO_2_ group, which can coordinate with Zn through electron-donating O and N atoms, thereby suppressing the oxidation of Zn (Zn → Zn^2+^) and stabilizing the adsorption layer on the metal surface. When incorporated into the Ppy matrix, 3Nisa forms a Ppy/3Nisa composite coating that improves the adhesion and protective performance of Ppy on the Zn substrate. The molecular structure of Ppy^43^ and the proposed mechanism of Ppy deposition on Zn^41^ are illustrated in Fig. 3. The lone electron pairs of the O atoms from the –OH and –COOH groups, together with the N atom of the –NO_2_ group, can interact with Zn^2+^ ions to form coordination bonds, contributing to the formation of a compact and stable coating structure.

**Fig. 4:**
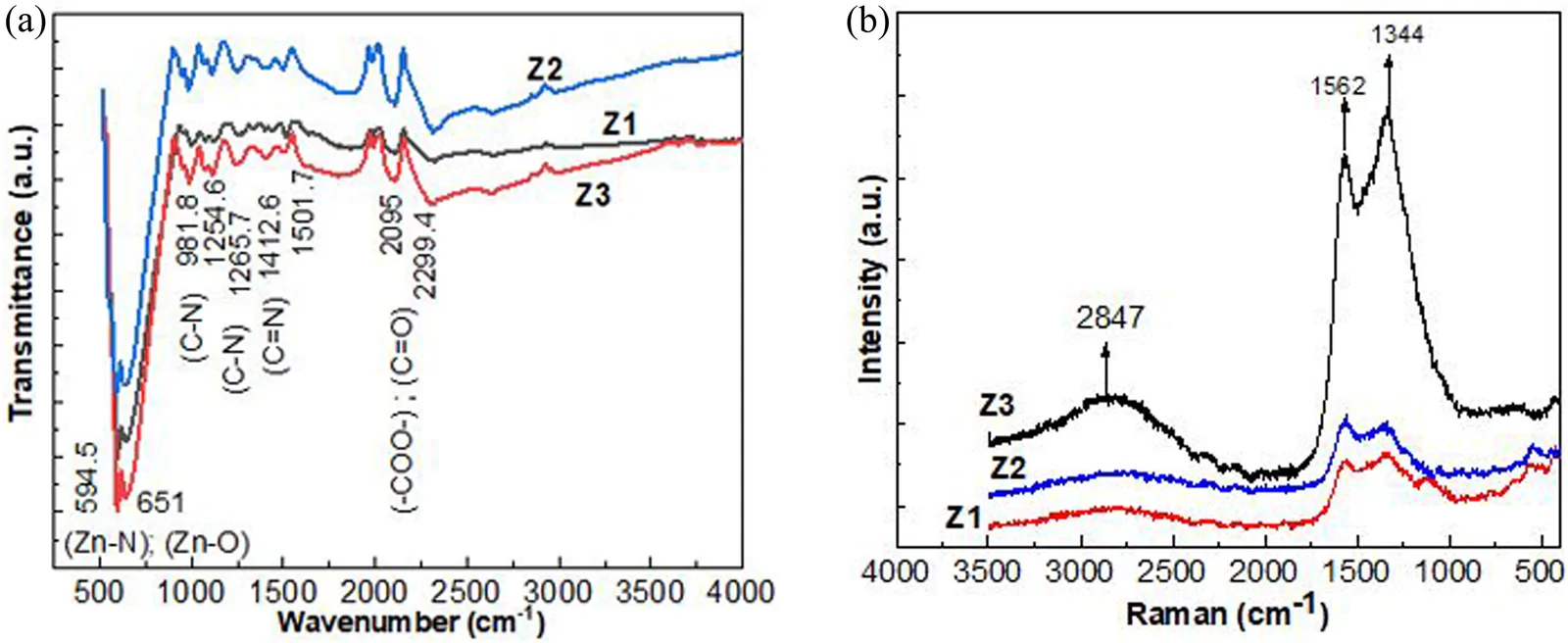
FT-IR (a) and Raman (b) spectra of the Ppy/3Nisa coatings on Zn prepared with different concentrations of 3Nisa (Z1, Z2, and Z3).

The FT-IR spectra of the samples are shown in Fig. 4(a). The characteristic absorption bands observed at 981.8, 1254.6, and 1265.7 cm^−1^ are assigned to the C–N stretching vibrations of the Ppy backbone. The bands located at 1412.6 and 1501.7 cm^−1^ are attributed to COO^−^ vibrations associated with the coordination of carboxylate groups from 3Nisa with Zn^2+^ ions. These peak positions are slightly shifted compared with the characteristic C=N vibrations of pure Ppy reported in the literature,^33^ indicating intermolecular interactions between Ppy, 3Nisa, and Zn ions.

In addition, the bands at 2095 and 2299.4 cm^−1^ are attributed to vibrations related to the –COO^−^ and C=O groups formed during pyrrole polymerization.^46^ The low-wavenumber bands at 594.5 and 651 cm^−1^ can be assigned to Zn–N and Zn–O coordination vibrations, respectively, confirming the formation of coordination bonds between Zn^2+^ ions and the N/O donor atoms of Ppy and 3Nisa. The presence of these coordination interactions suggests the formation of a metal–organic network involving Ppy, 3Nisa, and Zn^2+^ ions. Such a network is expected to enhance coating stability, improve Zn^2+^ immobilization, and contribute to the self-healing capability of the protective layer.

The Raman spectra of the samples are presented in Fig. 4(b). Consistent with the FT-IR findings, Raman analysis further confirms the successful formation of the Ppy/3Nisa composite coating and the interactions between Ppy, 3Nisa, and Zn^2+^ ions. The incorporation of 3Nisa into the Ppy matrix leads to the formation of a coordinated Ppy/3Nisa system on the Zn surface. Consequently, the Raman spectra of samples Z1, Z2, and Z3 exhibit characteristic vibrational bands comparable to those reported for Ppy in previous studies.^33^ The band located at 1344 cm^−1^ is attributed to the N–C stretching vibration of the pyrrole ring, while the band at 1562 cm^−1^ corresponds to the stretching vibration of the conjugated C–C=C backbone of Ppy. These characteristic peaks confirm the successful polymerization of pyrrole and the formation of a conductive Ppy structure within the composite coating. Moreover, slight shifts in position and larger changes in peak intensity with increasing 3Nisa content (sample Z3) may arise from the coordination interactions between Ppy, 3Nisa, and Zn^2+^ ions. Thus, the presence of 3Nisa can influence the electronic structure and conjugation length of the Ppy chains, resulting in modifications to the Raman vibrational features. Together with the FT-IR analysis, these results provide strong evidence for the formation of a coordinated metal–organic Ppy/3Nisa network on the Zn substrate, which contributes to improved coating stability and protective performance.

#### 3.2.3. Thermal stability of the coatings

**Fig. 5:**
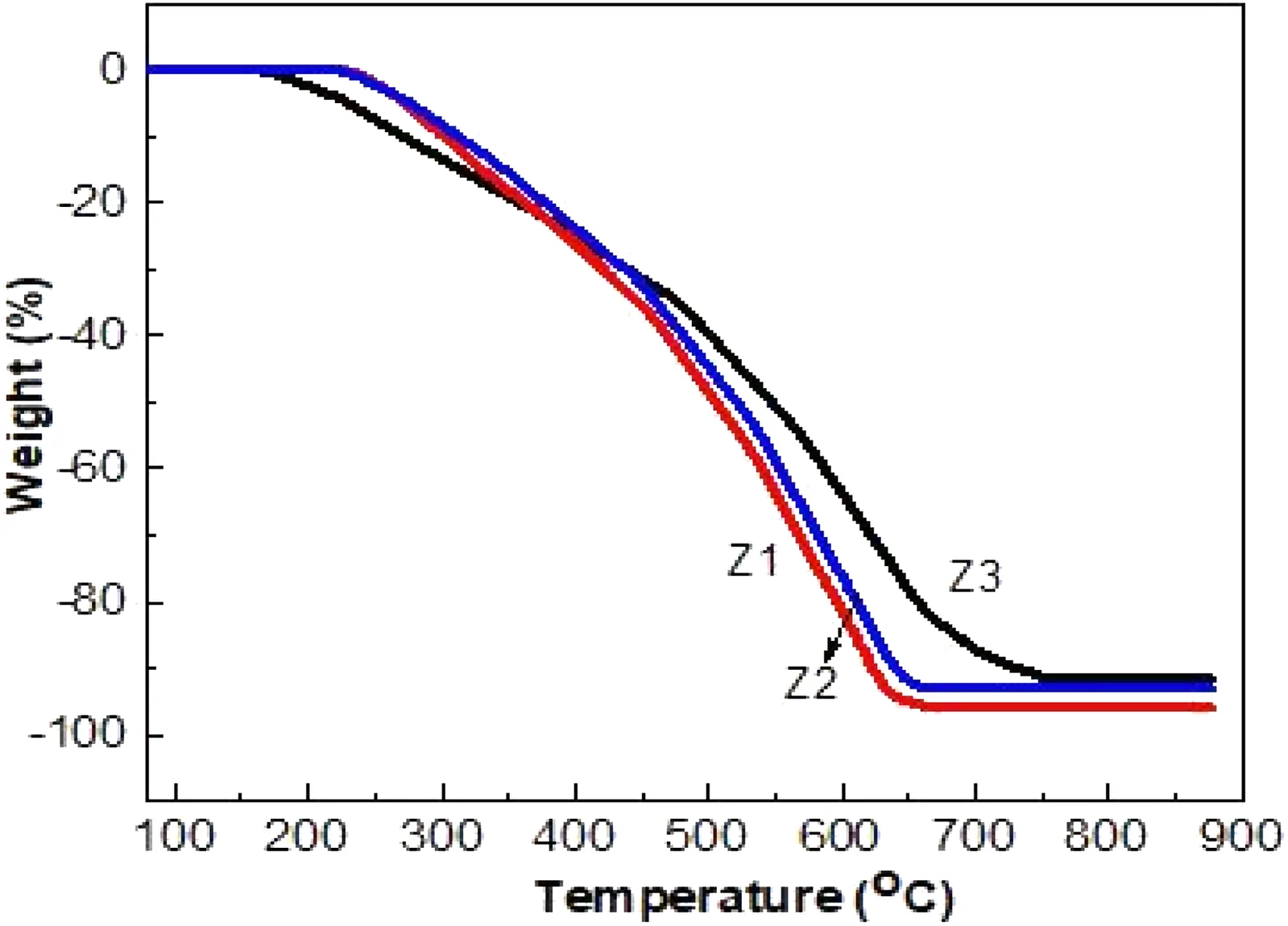
TGA curves of the Z1, Z2, and Z3 samples.

Thermogravimetric analysis (TGA) was conducted to investigate the effect of the 3Nisa dopant concentration (0.005, 0.01, and 0.02 M) on the thermal stability of the Ppy/3Nisa coatings deposited on Zn substrates, as shown in Fig. 5. All samples exhibited negligible weight loss below approximately 250 °C, which can be attributed to the removal of physically adsorbed water and residual solvent molecules.

The main decomposition stage occurred in the temperature range of 300 °C–650 °C, corresponding to the degradation of the polypyrrole backbone together with the decomposition of dopant-related species associated with 3Nisa.

Among the investigated samples, Z3, prepared with 0.02 M 3Nisa, exhibited the highest thermal stability, as indicated by the delayed onset of thermal decomposition, a lower mass-loss rate, and a higher residual mass at elevated temperatures. In contrast, sample Z2 (0.01 M 3Nisa) showed the lowest thermal stability, whereas sample Z1 (0.005 M 3Nisa) displayed intermediate thermal behavior. The enhanced thermal stability observed for sample Z3 may be attributed to the stronger intermolecular interactions between the polymer chains, improved structural compactness, and more effective incorporation of 3Nisa molecules into the Ppy matrix.

In addition, the coordination interactions between Zn^2+^ ions and the N/O-containing functional groups of Ppy and 3Nisa may contribute to the formation of a more stable metal–organic network structure, thereby improving the thermal resistance of the composite coating.

### 3.3. Electrochemical properties of Ppy/3Nisa on Zn

**Fig. 6:**
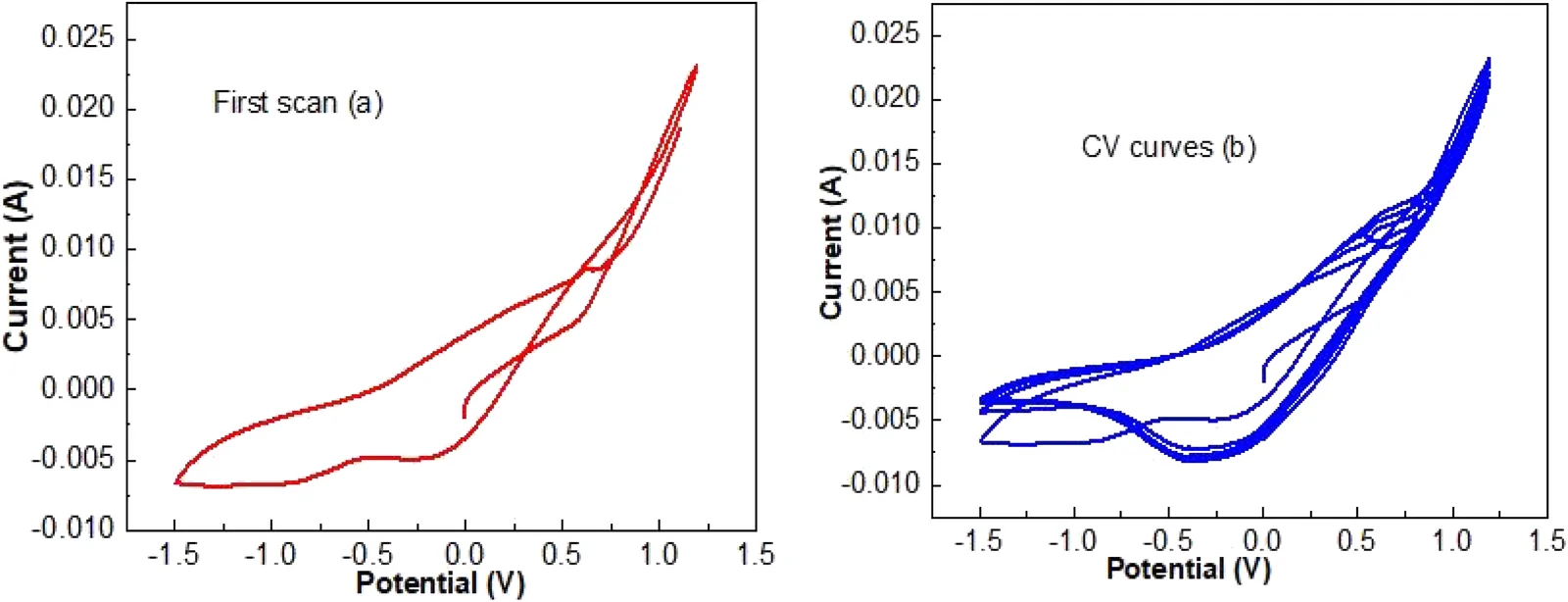
Cyclic voltammetric measurements of the Ppy/3Nisa/Zn sample in 0.1 M TBA at a rate of 20 mV s^−1^: (a) first scan and (b) CV curves.

Cyclic voltammetry was employed in this work to probe the redox behavior and electrochemical reversibility of the Ppy/3Nisa film deposited on Zn, rather than to directly evaluate its corrosion resistance. As polypyrrole is an electroactive conducting polymer, its oxidation/reduction behavior and ion-exchange capability are closely related to its coating functionality. A potential window ranging from −1.5 V to 1.2 V was selected to cover both the reduction and re-oxidation processes of the Ppy/3Nisa film in the TBA-Br electrolyte (Fig. 6).

To investigate the redox behavior of polypyrrole doped with 3-nitrosalicylic acid coated on zinc (Ppy/3Nisa/Zn), cyclic voltammetry (CV) measurements were performed in 0.1 M tetrabutylammonium bromide (TBA-Br) electrolyte at a scan rate of 20 mV s^−1^ (Fig. 6). In the presence of the TBA^+^ electrolyte, the bulky tetrabutylammonium cations cannot be incorporated into the Ppy matrix during reduction. Therefore, charge compensation occurs mainly through the expulsion of 3Nisa anions from the polymer film.^47^ As shown in Fig. 6(a), the Ppy/3Nisa film exhibits a reduction peak at approximately −0.25 V (*vs.* SCE) during the first cathodic scan. Upon reversing the scan direction, a re-oxidation peak appears at around 0.5 V (*vs.* SCE), which can be attributed to the incorporation of bromide anions from the electrolyte into the polymer matrix. The electrochemical response of the first cycle differs from that of the subsequent cycles. In later scans, the cathodic peak shifts toward more negative potentials and then remains nearly constant in subsequent cycles. The repetitive and stable voltammograms observed in Fig. 6(b) indicate that the Ppy/3Nisa film maintains good electrochemical reversibility and preserves its redox activity during repeated cycling.

These results suggest that 3Nisa anions are successfully incorporated into the Ppy matrix during electropolymerization and could subsequently be released during electrochemical reduction. Simultaneously, bromide anions from the electrolyte are incorporated into the polymer to maintain charge neutrality. Throughout the potential cycling process, the Ppy/3Nisa/Zn films retain their electrochemical properties without noticeable evidence of film degradation or decomposition.

To further understand the electrochemical behavior of the Ppy/3Nisa coating during reduction, EIS measurements were performed at different applied reduction potentials. This experiment was not intended to determine the polarization resistance in the conventional sense but rather to examine how the impedance response of the coating changes with the redox state of the polymer film. As the Ppy matrix is reduced, charge neutrality may be maintained by the release of incorporated dopant anions, which can influence the coating capacitance, ionic transport, and barrier properties. Therefore, the potential-dependent EIS results provide mechanistic information on the redox-state-dependent barrier behavior of the Ppy/3Nisa film and its possible relation to inhibitor release from the coating.

**Fig. 7:**
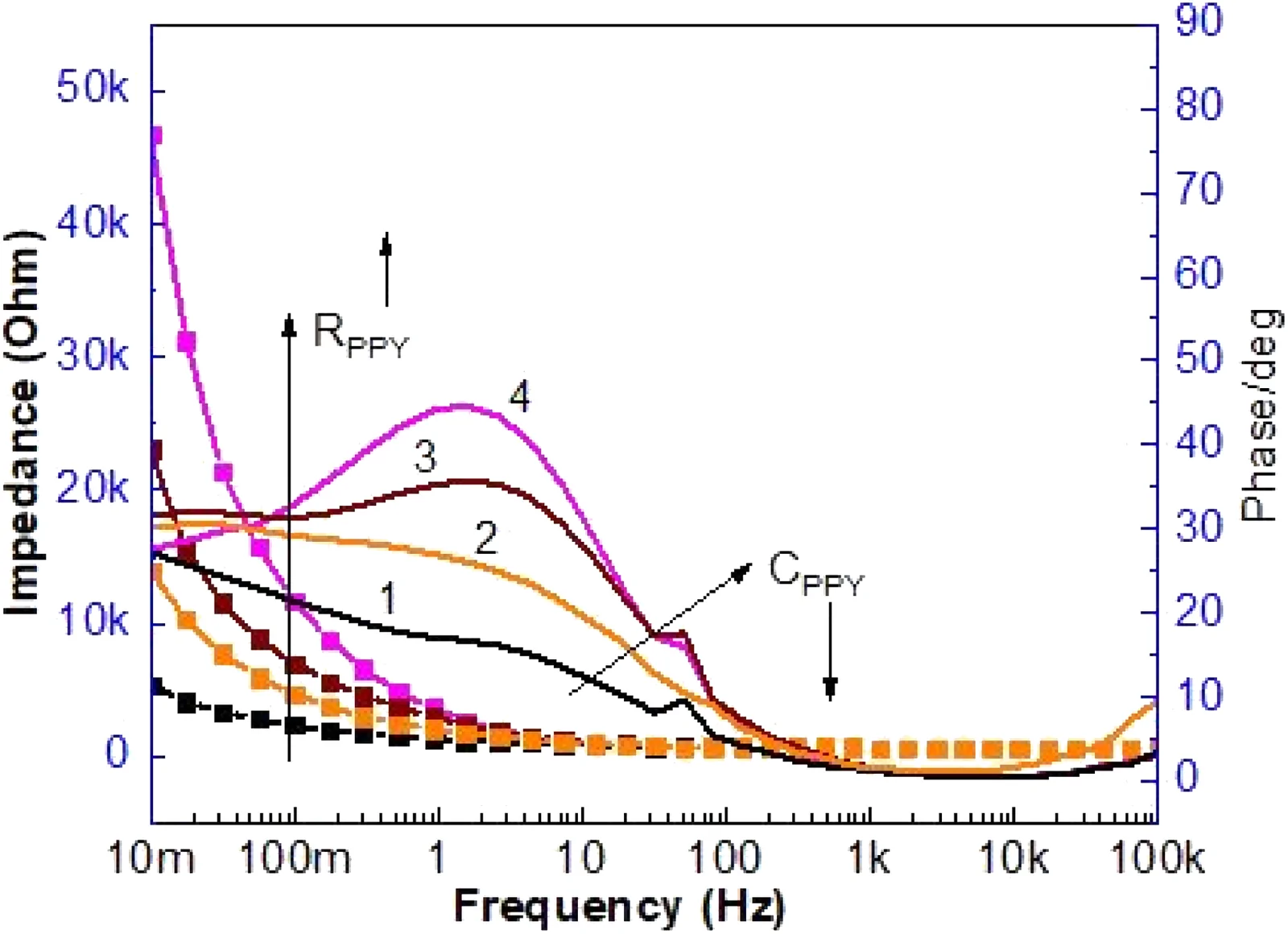
Bode plots of the Ppy/3Nisa/Zn (sample Z3) during reduction at (1) 0.2 V, (2) 0.4 V, (3) 0.6 V, and (4) 1.0 V *vs.* SCE.

Fig. 7 presents the potential-dependent Bode plots of the Ppy/3Nisa coating on Zn (representative sample Z3) immersed in a 3.5 wt% NaCl solution. Curves 1, 2, 3, and 4 correspond to reduction potentials of −0.2, −0.4, −0.6, and −1.0 V, respectively. An increase in the phase angle is observed with shifting reduction potentials in the frequency range of 100 mHz to 30 Hz. This indicates that the coatings remain non-destructive and exhibit predominantly capacitive behavior. Conversely, a decrease in the phase angle at the remaining frequencies reflects the penetration of corrosive electrolyte species into the coating and the increasing contribution of coating capacitance.^48^ Therefore, changes in the phase angle can be used to evaluate the anticorrosion performance of coatings.

As shown in Fig. 7, the low-frequency impedance value (|*Z*|_0.1_ Hz) of the Ppy/3Nisa coating increases, while the coating capacitance slightly decreases during the reduction process, indicating the release of 3Nisa anions from the polymer matrix. The results demonstrate that the Ppy film doped with 3Nisa provides an effective barrier against the diffusion of corrosive species toward the zinc surface.^49^ Furthermore, the relatively high mobility of 3Nisa anions may contribute to a self-healing effect, thereby enhancing the corrosion protection performance of the coating.

### 3.4. Corrosion test in a 3.5 wt% NaCl solution

**Fig. 8:**
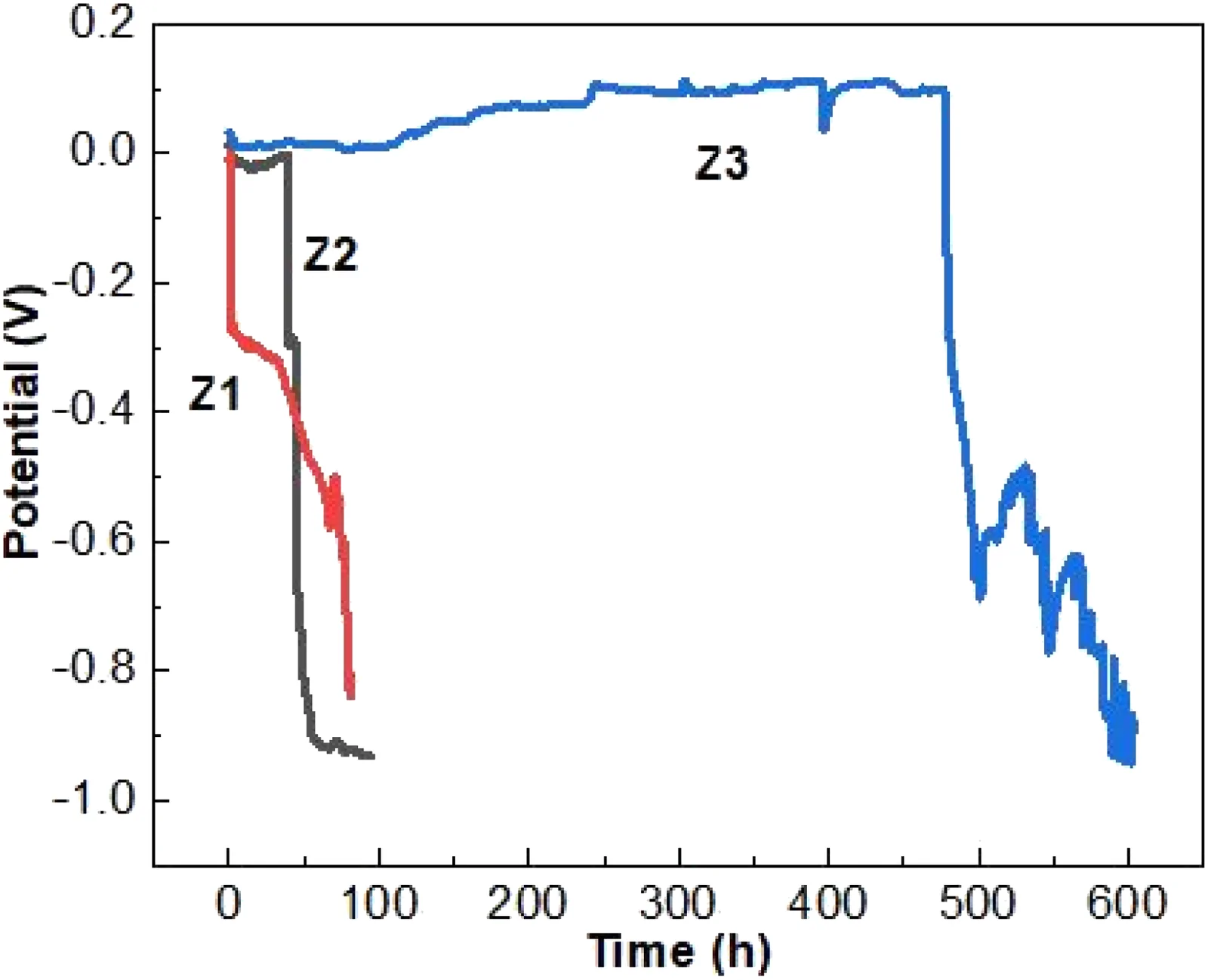
OCP-time curves of the Z1, Z2 and Z3 samples in a 3.5% NaCl solution.

The corrosion protection performance of the Ppy/3Nisa coatings on Zn substrates was evaluated by immersion in a 3.5 wt% NaCl solution. The open circuit potential (OCP) was monitored for samples prepared under different conditions (Z1, Z2, and Z3), as shown in Fig. 8. The Ppy/3Nisa-coated Zn samples initially exhibited relatively positive potentials in the range of 0 to −0.1 V, indicating that the Zn surface remained in a passive state for a prolonged period, particularly for samples Z2 and Z3. In contrast, the OCP of sample Z1 decreased immediately after immersion; however, the potential stabilized at approximately −0.3 V, which still corresponded to the passive region of Zn.

Among the investigated samples, Z3 exhibited the longest OCP plateau, lasting for approximately 500 h. The duration of this plateau can be considered an indicator of the corrosion protection efficiency of the coating. Notably, a second OCP plateau appeared after approximately 500 h of immersion. Before the OCP dropped to the corrosion potential of Zn (approximately −1.0 V), fluctuations in the potential were observed, suggesting ongoing electrochemical processes and the partial degradation of the protective layer. In addition, white corrosion products formed immediately on the bare Zn surface after exposure to the NaCl solution, indicating a severe corrosion attack.

The OCP of the Ppy/3Nisa coated Zn electrode should be interpreted as the mixed potential of the entire coating/substrate/electrolyte system. It results from the balance of all accessible anodic and cathodic reactions rather than from the equilibrium potential of Zn alone. Therefore, the relatively positive OCP values observed for the Ppy/3Nisa-coated samples, particularly Z2 and Z3, indicate that the coating effectively suppresses the overall electrochemical activity of the Zn surface and shifts the mixed potential toward a less active region. The prolonged OCP plateau observed for Z3 is thus regarded as evidence of the sustained protective behavior of the coated system during immersion although it should not be regarded as direct proof of a single passivation mechanism. Accordingly, the OCP results were analyzed together with the polarization, EIS, and salt spray data to evaluate the anticorrosion performance of the coatings. No visible precipitates were observed on the coated Zn samples, suggesting that the Zn surface remained passivated beneath the protective coating. These results demonstrate that Ppy doped with 3Nisa can effectively suppress the corrosion of Zn substrates with 0.02 M 3Nisa in the electropolymerization electrolyte (sample Z3), yielding superior corrosion protection compared with Z2 and Z1.

**Fig. 9:**
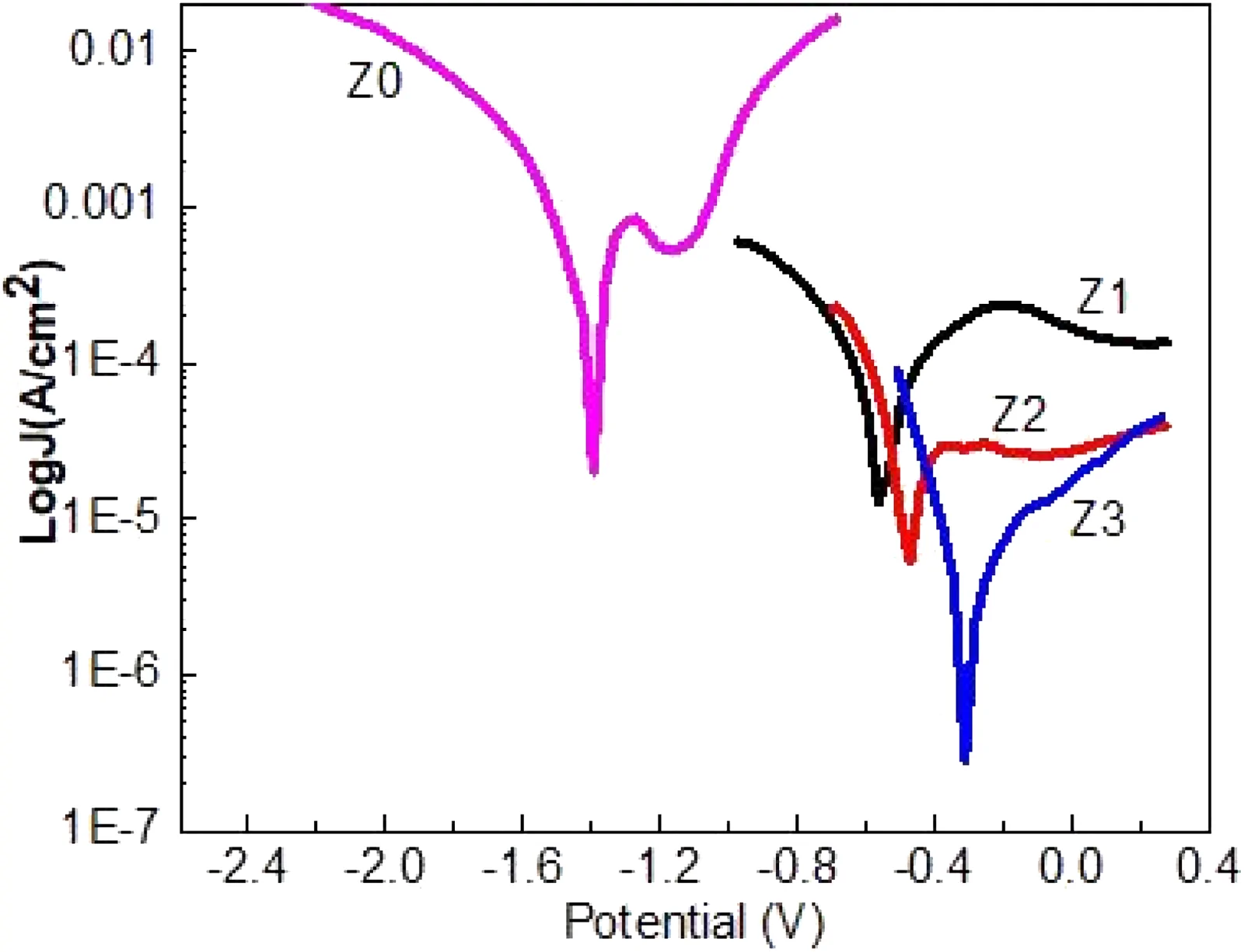
Tafel plots of the Z0, Z1, Z2 and Z3 samples in a 3% NaCl solution at a scan rate of 10 mV s^−1^.

To further investigate the corrosion protection mechanism, potentiodynamic polarization measurements were carried out using the linear potential sweep method at a scan rate of 10 mV s^−1^ in an aerated 3.5 wt% NaCl solution. The potential was scanned from −2.4 V to 0.4 V, and the corresponding polarization curves are presented in Fig. 9, together with those of bare Zn.

The corrosion parameters obtained from the Tafel extrapolation revealed that the corrosion current densities (*I*_corr_) of Z0, Z1, Z2, and Z3 were 108, 75.7, 7.99, and 1.47 µA cm^−2^, respectively. A significant decrease in *I*_corr_ was observed with increasing 3Nisa concentration in the coating, indicating an enhanced corrosion resistance. In particular, sample Z3 exhibited the lowest corrosion current density and the most positive corrosion potential, confirming its superior protective performance. The reduced corrosion current demonstrates that the incorporation of higher amounts of 3Nisa into the Ppy matrix effectively improves the anticorrosion properties of the coating.

The corrosion current densities were further used to calculate the corrosion inhibition efficiency (*η*) of the Ppy/3Nisa coatings according to eqn (1) as follows:1
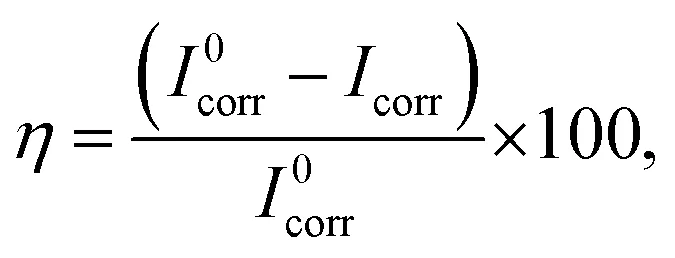
where *I*^0^_corr_ and *I*_corr_ are the corrosion current densities of the bare Zn and Ppy/3Nisa coated Zn, respectively. The corrosion inhibition efficiency of sample Z3 reached 98.6%, which was significantly higher than those of Z1 (29.9%) and Z2 (92.6%). Compared with previously reported dopants, as shown in Table 2, 3Nisa demonstrated excellent effectiveness as a dopant for enhancing the corrosion protection performance of Zn substrates.

**Table 2:** Comparison of the Ppy films on Zn for their protection efficiency

Reference	C. Pirvu *et al.*^50^	V. Brânzoi *et al.*^51^	This work
Substrate	Zn on Fe	Zn	Zn
Dopant	Tartaric acid	Dodecylbenzenesulfonate and 1,4-bis(2-ethylhexyl) sulfosuccinate	3Nisa
*E*_corr_ (V) *vs.* SCE	−0.395	−0.916	−0.302
*I*_corr_ (µA cm^−2^)	110	37	1.47
Protection efficiency	—	88.24%	98.6%

The improved corrosion resistance originates primarily from the reduction in the overall corrosion current due to the barrier effect of the coating and inhibitor-assisted repassivation. The cathodic branch represents the oxygen reduction and/or hydrogen evolution reactions associated with the corrosion process and should not be interpreted as evidence of a cathodic protection mechanism.

Upon reduction, the continuous Ppy/3Nisa film becomes cation-permselective, allowing only a limited amount of 3Nisa dopant anions to be released from the polymer matrix.^47^

**Fig. 10:**
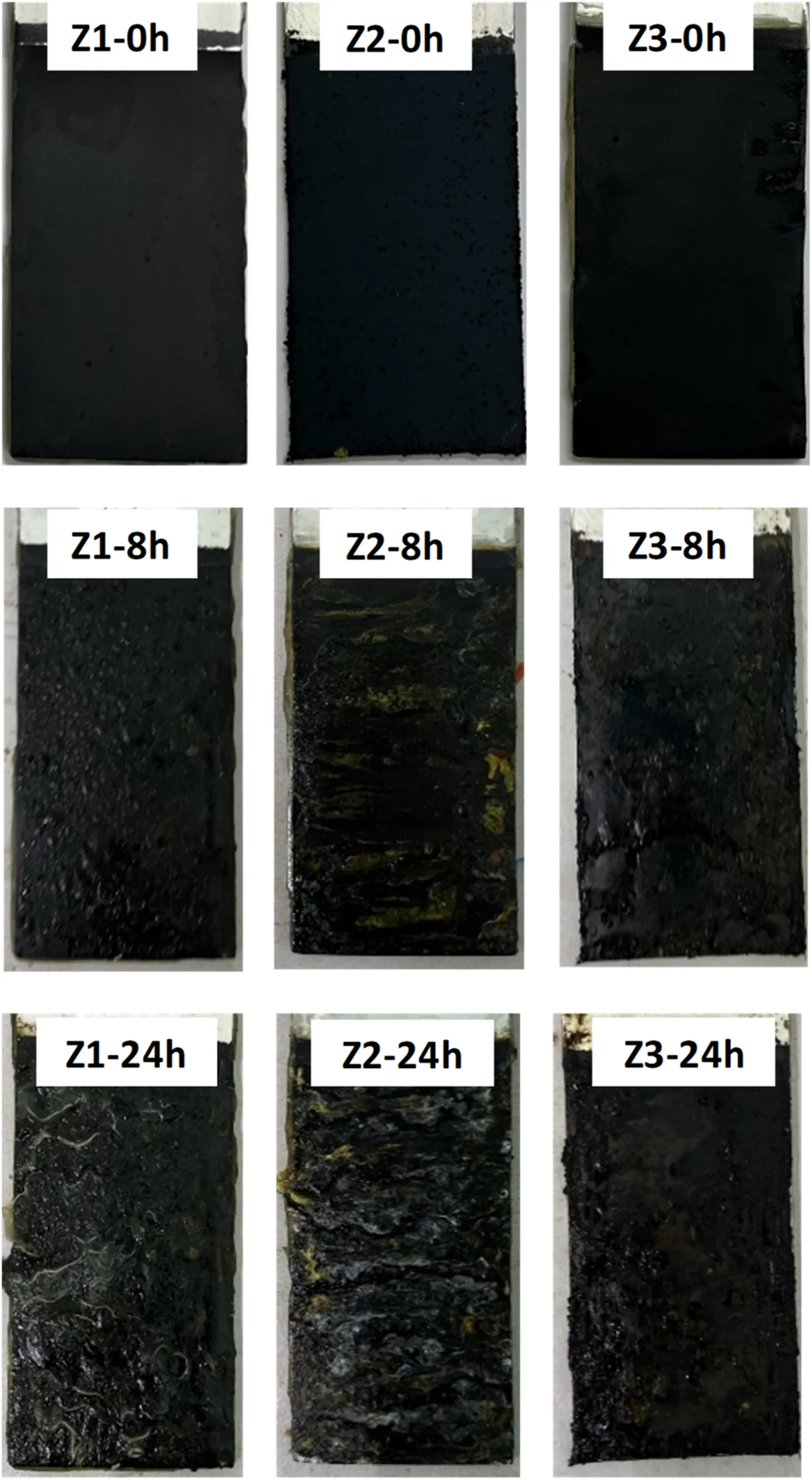
Salt spray corrosion tests of the Z1, Z2, and Z3 samples after 0, 8, and 24 hours of exposure.

The corrosion resistance of the Ppy/3Nisa coatings was further evaluated using a salt spray test. Samples Z1, Z2, and Z3 were exposed to salt spray conditions for 0 h, 8 h and 24 h, and the results are presented in Fig. 10.

The surface morphology of Ppy films doped with 3Nisa on Zn substrates revealed progressive degradation with increasing salt spray exposure time. After 8 h of exposure, the coatings generally retained relatively compact structures, especially sample Z3, which exhibited a dense and uniform morphology. In contrast, both samples Z1 and Z2 displayed rougher and more porous surfaces with the appearance of initial corrosion features. After 24 h, all samples showed increased structural deterioration; however, samples Z1 and Z2 suffered severe degradation and substantial accumulation of corrosion products, whereas sample Z3 maintained comparatively better structural integrity with fewer visible defects (Fig. 10). These observations indicate that the incorporation of 3Nisa improves the compactness of the Ppy coating and significantly enhances its corrosion resistance.

The aforementioned FT-IR analysis of 3Nisa and Ppy, together with the electrochemical and salt spray test results in a NaCl environment, confirmed that sample Z3 exhibited improved barrier properties, coating stability, and adhesion strength. This enhanced performance can be attributed to the formation of coordination interactions, such as Zn–N and Zn–O bonds, as well as the development of a denser coating structure. These features effectively suppress electrolyte penetration and Zn dissolution while simultaneously stabilizing Zn^2+^ ions and facilitating self-healing behavior through *in situ* repassivation processes.

The improved anticorrosion performance of the Ppy/3Nisa coating is attributed to the synergistic effects of enhanced barrier properties, stronger interfacial interactions between the coating and Zn substrate, and the possible release of inhibitive 3Nisa species during electrochemical reduction. Based on the electrochemical behavior of the Ppy/3Nisa film and the effectiveness of corrosion resistance, this process may contribute to an inhibitor-assisted repassivation or self-healing-like effect at locally activated sites. However, the current findings should be considered indirect evidence of such a mechanism, and further time-resolved EIS and post-corrosion surface analysis are required for direct confirmation.

Although the obtained SEM, FT-IR, Raman, TGA, and electrochemical results consistently support the formation of protective Ppy/3Nisa coatings on Zn, additional characterization would further strengthen the structural and mechanistic interpretations. In particular, cross-sectional SEM and EDS elemental mapping provide direct information on the coating thickness, interfacial continuity, and dopant-related elemental distribution across the film. Likewise, the post-corrosion characterization of salt-spray-exposed surfaces is highly beneficial for identifying corrosion products and clarifying the relationship between coating degradation, inhibitor release, and repassivation behavior. These aspects will be addressed in future studies.

It should be emphasized that the present Ppy/3Nisa coating does not function through a classical cathodic protection mechanism. In conventional corrosion systems, cathodic reactions involve oxygen reduction in aerated electrolytes or hydrogen evolution under acidic conditions. In contrast, the electrochemical behavior observed in this work mainly reflects the barrier characteristics of the conductive polymer coating, together with inhibitor-assisted repassivation and coordination interactions between Zn, Ppy, and 3Nisa. Consequently, the improved corrosion resistance results from the suppression of both anodic metal dissolution and overall corrosion kinetics rather than from cathodic protection.

## 4 Conclusions

Adherent and homogeneous Ppy/3Nisa coatings were successfully electrodeposited on Zn substrates by a simple one-step electrochemical method with pyrrole in a 3-nitrosalicylic acid solution. SEM, FT-IR, and Raman analyses confirmed the successful formation of compact Ppy/3Nisa films on the Zn surface.

Electrochemical measurements, including cyclic voltammetry, OCP, EIS, and polarization studies, demonstrated that the Ppy/3Nisa coatings significantly enhanced the corrosion resistance of Zn in a 3.5 wt% NaCl solution. Among the investigated samples, Z3 exhibited the best performance, with the lowest corrosion current density and a corrosion inhibition efficiency of 98.6%. The improved protection is attributed to the synergistic effects of barrier protection, Zn–N/Zn–O coordination interactions, and controlled release of 3Nisa inhibitor species, which facilitate inhibitor-assisted repassivation. No evidence supporting a cathodic protection mechanism was observed under the present experimental conditions.

Salt spray tests further confirmed that the Ppy/3Nisa coatings, particularly Z3, provided superior structural stability and corrosion protection in aggressive saline environments. These results demonstrate that 3Nisa is an effective dopant for improving the anticorrosion performance of Ppy coatings on Zn substrates and may offer potential applications in Zn-based protective and energy storage systems.

## Conflicts of interest

There are no conflicts to declare.

## Data Availability

The datasets used and/or analyzed during the current study are included in the article.

## References

[cit1] Bazli L., Yusuf M., Farahani A., Kiamarzi M., Seyedhosseini Z., Nezhadmansari M., Aliasghari M., Iranpoor M. (2020). J. Compos. Compd..

[cit2] Zadeh M. K., Yeganeh M., Shoushtari M. T., Esmaeilkhanian A. (2021). Synth. Met..

[cit3] Yin Y., Prabhakar M., Ebbinghaus P., Silva C. C., Rohwerder M. (2022). Chem. Eng. J..

[cit4] Kumar A. (2023). World J. Adv. Res. Rev..

[cit5] Mametja S., Mmelesi O. K., Sefadi J. S., Liu X., Gorimbo J. (2025). J. Power Sources.

[cit6] Ni Y., Liu Q., Xue T., Zang L., Yu X., Zhang J., Yang C. (2025). Int. J. Biol. Macromol..

[cit7] Khamsanga S., Uyama H., Nuanwat W., Pattananuwat P. (2022). Sci. Rep..

[cit8] Zhu Q., Li E., Liu X., Song W., Zhao M., Zi L., Wang X., Liu C. (2020). Composites, Part A.

[cit9] Peng C., Zhang Y., Yang S., Zhang L. L., Wang Z. (2022). Nano Energy.

[cit10] Luo H., Li C., Sheng W., Yuan W., Chen X., Ma Q., Li X., Sun Z., Li P. (2025). Solid State Ionics.

[cit11] Xu S., Sun Z., Wei D., Shao L., Wang M., Wang Z., Zhang J., Zhang T., Liu G., Cui J., Wei M., Dong Y. (2024). J. Membr. Sci..

[cit12] Zhang R., Song M., Zhang W., Shao A., Zhang L., Zhang H., Chao D., Zhou W. (2025). ACS Appl. Mater. Interfaces.

[cit13] Ma Y., Li T., Zhao S. R., Feng Z. T., Liu J. K. (2025). Nano Res..

[cit14] Asan G., Asan A., Çelikkan H. (2020). J. Mol. Struct..

[cit15] Zhang Y., Die J., Li F., Li H., Tu J., Zhang K., Yu X. (2023). Coatings.

[cit16] Jaouhari A. E., Chennah A., Jaddi S. B., Ahsaine H. A., Anfar Z., Alaoui Y. T., Naciri Y., Benlhachemi A., Bazzaoui M. (2019). Surf. Interfaces.

[cit17] Zhu H., Liu X., Hao H., Zheng X. (2022). Metals.

[cit18] Muresan L. M. (2023). Materials.

[cit19] Tsuchiya S., Ueda M., Ohtsuka T. (2007). ISIJ Int..

[cit20] Bazzaoui M., Martins J. I., Bazzaoui E. A., Reis T. C., Martins L. (2004). J. Appl. Electrochem..

[cit21] Branzoi V., Pruna A., Branzo F. (2008). Mol. Cryst. Liq. Cryst..

[cit22] Ryu H., Sheng N., Ohtsuka T., Fujita S., Kajiyama H. (2012). Corros. Sci..

[cit23] Vu Q. T., Pham V. H., Duong Q. P., Le M. D., Le T. T. H. (2012). J. Exp. Nanosci..

[cit24] Hermelin E., Petitjean J., Lacroix J. C., Chane-Ching K. I., Tanguy J., Lacaze P. C. (2008). Chem. Mater..

[cit25] Martins J. I., Reis T. C., Bazzaoui M., Bazzaoui E. A., Martins L. (2004). Corros. Sci..

[cit26] Ren Y., Chen J., He J., Zeng C. (2014). Key Eng. Mater..

[cit27] Rikhari B., Mani S. P., Rajendran N. (2016). RSC Adv..

[cit28] Tallman D. E., Spinks G., Dominis A., Wallace G. G. (2002). J. Solid State Electrochem..

[cit29] Xu J., Zhang Y., Tang Y., Cang H., Jing W. (2014). Ind. Eng. Chem. Res..

[cit30] Bazli L., Yusuf M., Farahani A., Kiamarzi M., Seyedhosseini Z., Nezhadmansari M., Aliasghari M., Iranpoor M. (2020). J. Compos. Compd..

[cit31] Bai Y., Jin X., Xie J., Lv X., Guo T., Zhang L., Zhu J., Shao Y., Zhang H., Zhang H., Yuan B., Yin A., Nie J., Cao F., Xu Z. (2022). Coatings.

[cit32] Guo Y., Jia S., Qiao L., Su Y., Gu R., Li G., Lian J. (2020). Colloids Surf., B.

[cit33] Vu Q. T., Trinh H. H., Tran H. Q., Ha M. H., Duong K. L., Hoang T. T. L., Le M. D. (2018). Corros. Eng., Sci. Technol..

[cit34] Ha M. H., Tran M. T., Le V. K., Le M. D., Hoang T. T. L., Lai T. H., Vu T. X., Nguyen T. B. V., Ngo X. L., Nguyen T. C., Thai H., Vu T. H., Vu Q. T. (2023). Des. Monomers Polym..

[cit35] Ha M. H., Duong K. L., Nguyen T. C., Le M. D., Vu Q. T. (2019). Prog. Org. Coat..

[cit36] Vu Q. T., Ha M. H., Le V. K., Le M. D., Nguyen T. B. V., Duong K. L., Vu T. H., Nguyen D. D., Doan T. Y. O., Ngo X. L., Nguyen T. C., Hoang T., Hoang T. T. L., Cao L. V., Ţălu Ş., Nguyen Trong D. (2022). ACS Omega.

[cit37] Chen Z., Yang W., Xu B., Guo Y., Chen Y., Yin X., Liu Y. (2018). Prog. Org. Coat..

[cit38] Zhao J., Qin H., Miao B., Zhang P., Zeng H., Wang D., Yuan G., Qian J., Chen Y. (2025). J. Magnesium Alloys.

[cit39] Bairagi H., Vashishth P., Ji G., Shukla S. K., Ebenso E. E., Mangla B. (2024). Corros. Commun..

[cit40] Baek J., Choi J. I., Kim S., Kim S., An G. H., Jang S. S. (2025). ACS Appl. Energy Mater..

[cit41] Zhang F., Wang C., Pan J., Tian F., Zeng S., Yang J., Qian Y. (2020). Mater. Today Energy.

[cit42] Kim S., Baek J., Choi J. I., Jo Y. R., Jang S. S., An G. H. (2025). Nano Energy.

[cit43] Zadeh M. K., Yeganeh M., Shoushtari M. T., Esmaeilkh A. (2021). Synth. Met..

[cit44] Dhachanamoorthi N., Jothi M., Tamilselvan S. (2020). Int. J. Sci. Technol. Res..

[cit45] Kancharla H., Mandal G. K., Maharana H. S., Singh S. S., Mondal K. (2023). J. Mater. Eng. Perform..

[cit46] Luo W., Qi K., Qiu Y., Guo X. (2023). Prog. Org. Coat..

[cit47] Yin Y., Prabhakar M., Ebbinghaus P., Silva C. C., Rohwerder M. (2022). Chem. Eng. J..

[cit48] Rammelt U., Duc L. M., Plieth W. (2005). J. Appl. Electrochem..

[cit49] Paliwoda-Porebska G., Rohwerder M., Stratmann M., Rammelt U., Duc L. M., Plieth W. (2006). J. Solid State Electrochem..

[cit50] Pirvu C., Mindroiu M., Demetrescu I. (2009). Key Eng. Mater..

[cit51] Branzoi V., Pruna A., Branzoi F. (2008). Mol. Cryst. Liq. Cryst..

